# KLF2– A Negative Regulator of Pre-B Cell Clonal Expansion and B Cell Activation

**DOI:** 10.1371/journal.pone.0097953

**Published:** 2014-05-29

**Authors:** Rebecca Winkelmann, Lena Sandrock, Jörg Kirberg, Hans-Martin Jäck, Wolfgang Schuh

**Affiliations:** 1 Division of Molecular Immunology, Department of Internal Medicine III, Nikolaus-Fiebiger-Center, University of Erlangen-Nürnberg, Erlangen, Germany; 2 Division of Immunology, Paul-Ehrlich-Institut, Langen, Germany; University Medical Center of the Johannes Gutenberg University of Mainz, Germany

## Abstract

Maturation as well as antigen-dependent activation of B cells is accompanied by alternating phases of proliferation and quiescence. We and others have previously shown that Krüppel-like factor 2 (KLF2), a regulator of T cell quiescence and migration, is upregulated in small resting precursor (pre)-B cells after assembly of the immature pre-B cell receptor (pre-BCR) and is downregulated upon antigen-induced proliferation of mature B cells. These findings suggest that KLF2, besides its function in maintaining follicular B cell identity, peripheral B cell homeostasis and homing of antigen-specific plasma cells to the bone marrow, also controls clonal expansion phases in the B cell lineage. Here, we demonstrate that enforced expression of KLF2 in primary pre-B cells results in a severe block of pre-BCR-induced proliferation, upregulation of the cell cycle inhibitors p21 and p27 and downregulation of c-myc. Furthermore, retroviral KLF2 transduction of primary B cells impairs LPS-induced activation, favors apoptosis and results in reduced abundance of factors, such as AID, IRF4 and BLIMP1, that control the antigen-dependent phase of B cell activation and plasma cell differentiation. Hence, we conclude that KLF2 is not only a key player in terminating pre-B cell clonal expansion but also a potent suppressor of B cell activation.

## Introduction

Krüppel-like factor 2 (KLF2/LKLF) belongs to the family of Krüppel-like transcription factors that bind to GC-rich DNA domains via three C-terminal zinc fingers and controls proliferation and terminal differentiation of various cell types [1]. KLF2 was originally discovered in lung tissue and was shown to be important for cardiovascular and lung development [2], [3], [4]. KLF2 also plays an important role in the development, activation and migration of T lymphocytes [5], [6], [7], [8], [9], [10], [11], [12]. During T cell development, KLF2 is upregulated in single-positive T cells and downregulated once these cells are activated, which suggests that KLF2 is an important regulator of quiescence in T cells [8]. Indeed, enforced expression of KLF2 in T cells results in inhibition of proliferation, which is mediated by upregulation of cell cycle inhibitor p21 and repression of c-myc [13], [14]. In B lymphocytes, KLF2 is induced as a consequence of pre-BCR signaling, and its expression is maintained until mature B cells are activated [15], [16], [17]. Additionally, high amounts of KLF2 transcripts were observed in anergic B cells, plasma cells as well as memory B cells, suggesting that KLF2 plays a role in maintaining B cell quiescence [18], [19], [20]. However, KLF2 deficiency in B cells has no impact on proliferation but results in an increase of marginal zone (MZ) B cells, a loss of peritoneal B1 cells and a defective homing of plasma cells to the bone marrow, presumably by regulating the expression of β7 integrin and CD62L [15], [17], [21]. Because loss of KLF2 in B cells has no impact on proliferation *in vivo*, it is tempting to speculate that other factors, such as KLF4, a closely related paralog of KLF2, compensate for KLF2 deficiency and take over cell cycle regulation. KLF4 not only shows an expression pattern similar to KLF2 but also shares overlapping target genes with KLF2, such as the cell cycle regulators p21 and c-myc [1]. To dissect the specific functions of KLF2 on proliferation, apoptosis and differentiation in B lymphoid cells, we performed enforced expression studies in freshly isolated pre-B cells and mature B cells using a retroviral expression construct for KLF2. In summary, retrovirally transduced KLF2 leads to increased levels of the cell cycle inhibitor proteins p21 and p27 and decreased levels of c-myc, resulting in a severe block of pre-BCR-induced proliferation. KLF2 is expressed in mature, resting B cells, downregulated in activated B cells and reexpressed in memory B cells [17], [18], [19], [20]. Retroviral transduction of LPS-stimulated primary splenic B cells with KLF2 resulted in inhibition of LPS-induced activation. KLF2 expression leads to a decline in IRF4, BLIMP1 and AID levels in transduced splenic B cells, suggesting that ectopic KLF2 expression interferes with the activation of mature B cells in response to LPS. Moreover, overexpression of KLF2 triggers the onset of apoptosis. Hence, we conclude that upregulation of KLF2 in late pre-B cells contributes to termination of pre-BCR-induced proliferation. Moreover, KLF2 counteracts LPS-induced activation, represses AID, IRF4 and BLIMP1 expression and is therefore critical to maintain mature B cells in a resting state.

## Results

### Enforced KLF2 Expression Inhibits pre-BCR-mediated Proliferation

We identified KLF2 as a candidate gene involved in the termination of pre-B cell clonal expansion [16]. To determine the functional role of KLF2 in the termination of pre-BCR-mediated clonal expansion, we used CD19^+^ B-lymphoid precursor cells from pre-BCR inducible animals (dTg) as an experimental model (Figure S1A in File S1) [16], [22], [23]. This model allows us to specifically induce pre-BCR expression and pre-BCR-mediated clonal expansion by tetracycline. In brief, we isolated µHC-negative CD19^+^ pro-B cells from the bone marrow of dTg animals by *MACS* cell sorting, and µHC/pre-BCR expression as well as pre-BCR-mediated proliferation was induced in the absence of tetracycline (Tet) in IL-7 cultures *in vitro* (Figure S1A in File S1).

**Figure 1:**
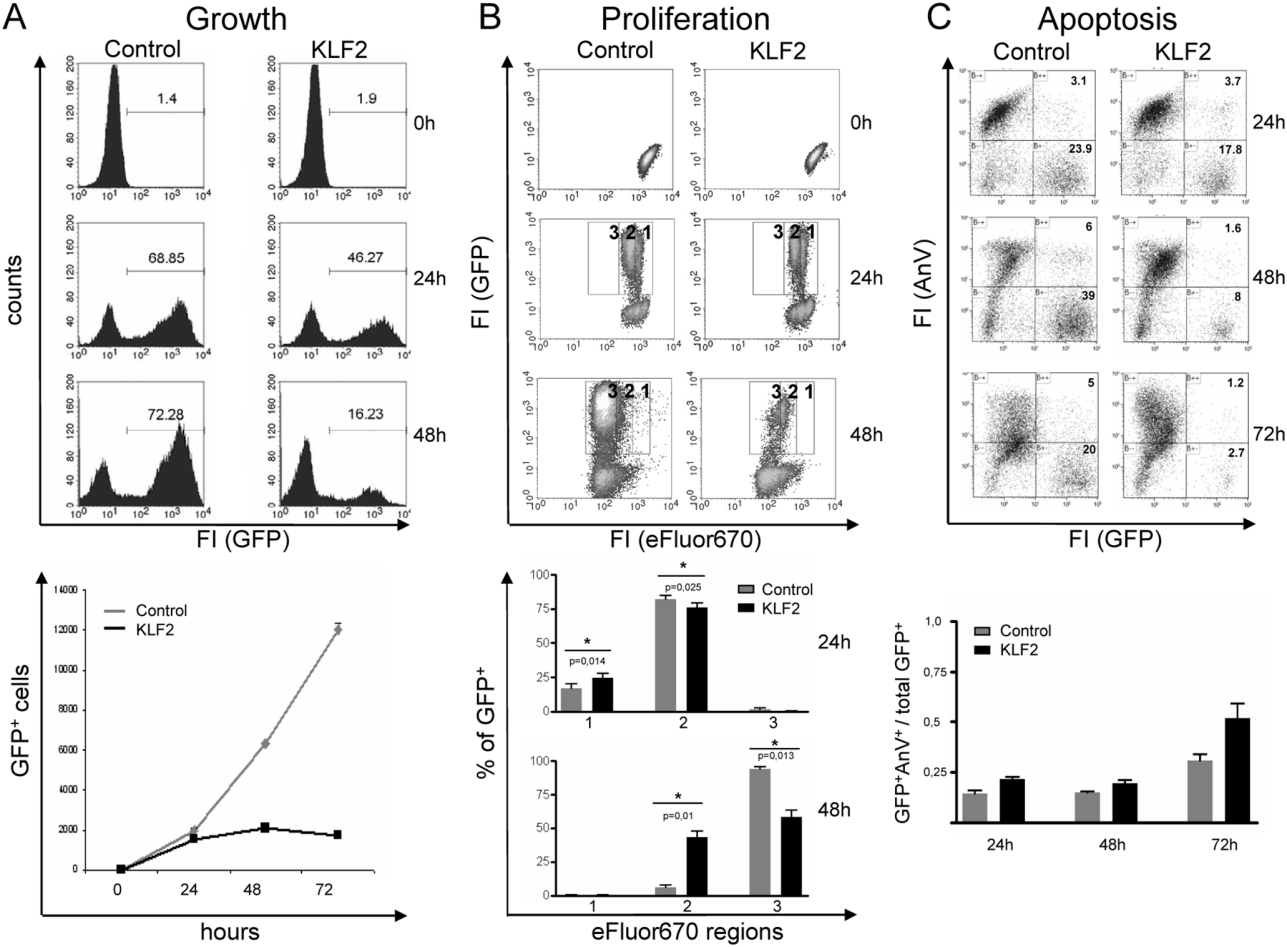
Enforced KLF2 expression inhibits the proliferation of pre-B cells. (**A–C**) Flow cytometric analyses of control- and KLF2-transduced primary CD19^+^ cells isolated from dTg animals. (**A**) Histograms show the frequencies of GFP^+^ cells (upper panel), the diagram (lower panel) shows the mean numbers ± SD of GFP-positive cells before (0) and 24 h–72 h after infection of one representative of 5 independent experiments measured in triplicate. (**B**) eFluor670-labeled GFP^+^ cells where analyzed for the loss of the proliferation dye (regions 1, 2 and 3) before (0) and 24 as well as 48 h after infection, with only GFP^+^ cells shown (upper panel). Bar diagrams show the mean frequencies ± SEM of GFP^+^ cells in the different boxes at the indicated time points and compare KLF2 (black)- and control (grey)-infected cells (n = 3) (lower panel). (**C**) Flow cytometric analyses of AnnexinV (AnV) and GFP fluorescence. Dot plots of one representative experiment (24, 48 and 72 h) are shown (upper panel). Numbers indicate frequencies of cells in the respective quadrants. Statistics show the mean ratios ± SD of AnnexinV^+^GFP^+^ vs. total GFP^+^ of pooled precursor B cells from 11 dTg animals measured in triplicate (lower panel).

**Figure 2:**
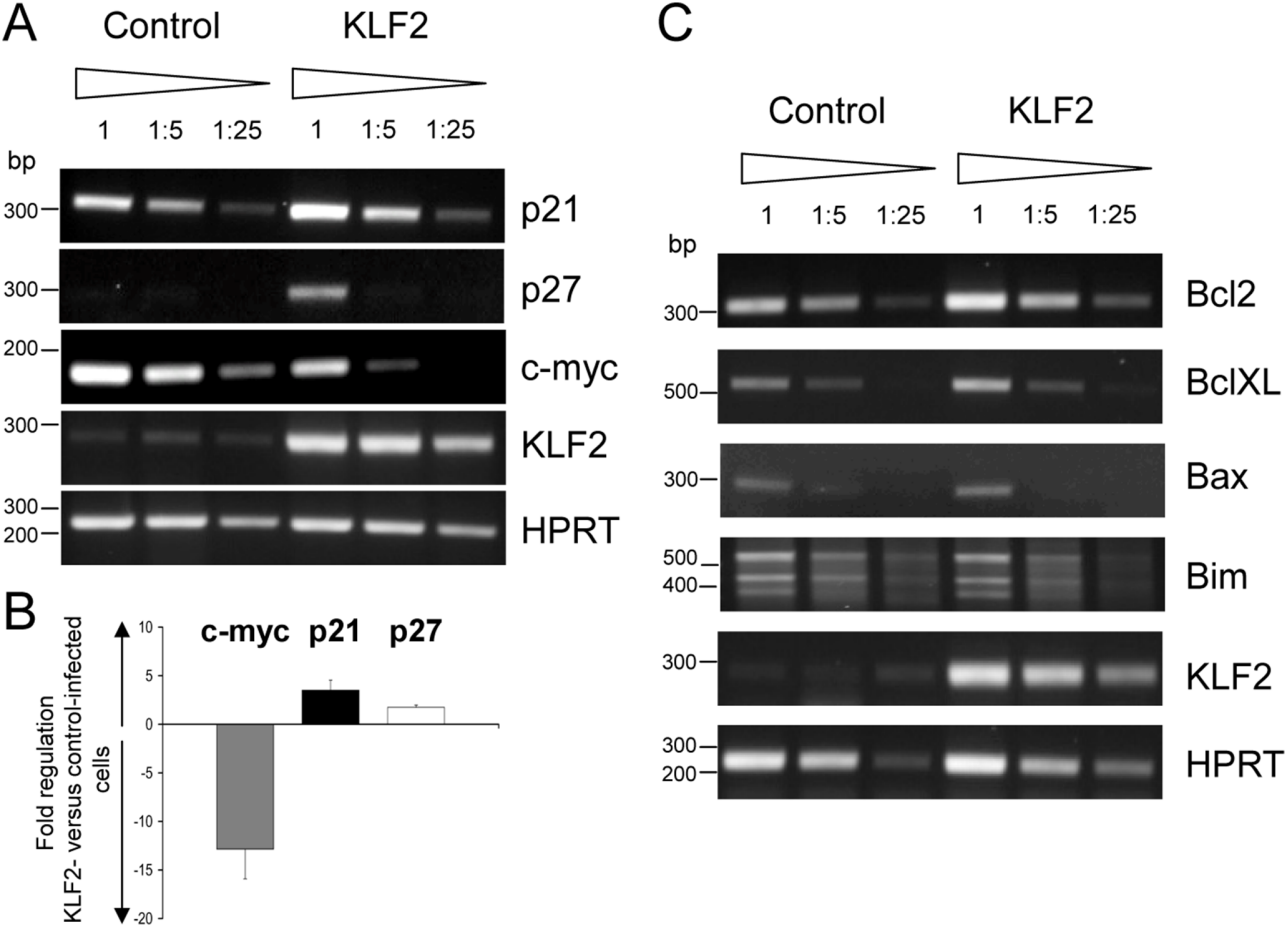
Enforced KLF2 expression induces p21 and p27 and represses c-myc. PCR analyses of cell cycle (**A, B**) and apoptotic regulators (**C**) in control- and KLF2-infected GFP^+^ sorted primary CD19^+^ bone marrow cells isolated from dTg animals 24 h after infection using transcript-specific primers. HPRT signals control the integrity and amount of cDNA. (**A, C**) Serial dilutions of the respective cDNAs were used as templates. Fragments of a DNA bp standard are indicated to the left. Representative results of three independent experiments are shown. (**B**) qPCR analyses of c-myc, p21 and p27 transcript levels. Bar graphs indicate the mean fold change (+/− SEM) of the respective transcript in KLF2-infected GFP^+^ compared to control-infected GFP^+^ CD19^+^ bone marrow cells from dTg animals 24 h after infection. The fold change of transcript abundance was calculated from four independent experiments.

To determine the effect of enforced KLF2 expression on pre-BCR-mediated proliferation, we retrovirally transduced primary CD19^+^ cells from dTg animals cultured in the absence of Tet (i.e., pre-BCR expression is turned on) with control (pBMN-IRES-GFP) and KLF2 (pBMN-KLF2–IRES-GFP) viral particles 24 h after isolation (Figures S1B, S2A in File S1). Successful infection was determined by flow cytometric analyses of GFP fluorescence, showing an infection rate of up to 70% (Figure 1A). Enforced KLF2 expression was confirmed by RT-PCR (Figure 2) and Western blotting (Figure S2B in File S1). To determine whether KLF2 transduction affects pre-BCR-induced cell growth, the numbers as well as frequencies of GFP^+^ cells were measured 24 h and 48 h after infection (Figure 1A). Analysis of GFP^+^ frequencies revealed that the frequencies as well as absolute numbers of KLF2-transduced cells strongly decreased from 24 h to 48 h after infection, whereas control virus-infected cells showed constant frequencies of GFP^+^ cells and an increase in the absolute numbers of GFP^+^ cells over time (Figure 1A). The numbers of KLF2-infected cells remained constant, indicating that enforced KLF2 expression blocks proliferation (Figure 1A, lower panel).

To assess the effect of KLF2 overexpression on proliferation, infected cells were labeled with eFluor670 proliferation dye and analyzed for eFluor670 fluorescence 0 h, 24 h and 48 h after retroviral infection using flow cytometry. To quantify the proliferation-dependent loss of the eFluor670 dye, the fluorescence intensities were staged into 3 different regions (Figure 1B, region 1–3: region 1, high eFluor670 intensities, to region 3, low eFluor670 intensities). Forty-eight hours upon infection, most of the control-infected cells (94%) were continuously growing and consequently lost the eFlour670 dye (region 3, Figure 1B), whereas KLF2-transduced cells showed higher levels of eFlour670 fluorescence. After 48 h, only 58% of the KLF2-transduced cells, compared with 94% of the control-infected cells, were in region 3 (Figure 1B, lower panel). Forty-two percent of KLF2-transduced cells still remained in region 2, compared to only approximately 5% of the control cells. Therefore, enforced expression of KLF2 in pre-B cells impairs cell cycle progression. To investigate whether KLF2-transduced cells undergo apoptosis, AnnexinV staining was performed and analyzed by flow cytometry. As shown in Figure 1C, there was no difference in the ratio of AnnexinV^+^GFP^+^ versus total GFP^+^ cells after 24 h and 48 h post infection. Upon 72 h there was a minor, but not significant increase in apoptosis. Taken together, enforced KLF2 expression results in a block of proliferation but has no significant impact on apoptosis in pre-B cells.

### KLF2 Blocks pre-BCR Induced Cell Growth by Regulating P21, P27 and c-myc

To investigate how KLF2 interferes with cell cycle progression, we analyzed the transcript abundance of cell cycle regulators such as p21, p27 and c-myc in control- and KLF2-transduced dTg pre-B cells by RT-PCR. Twenty-four hours upon infection with control and KLF2 viral supernatants, GFP^+^ dTg pre-B cells were sorted using a MoFlo cell sorter, and RNA was isolated (experimental setup: see Figures S1 and S3 in File S1). RT-PCR as well as qPCR analyses revealed that enforced KLF2 expression leads to an upregulation of the cell cycle inhibitors p21 (3.5 fold) and p27 (1.7 fold) and to a massive downregulation of c-myc (12.8 fold) (Figures 2A and 2B). In addition, we found that the transcript levels of apoptotic regulators such as Bcl2, BclXL, Bax and Bim were not altered (Figure 2C), indicating that enforced KLF2 expression in pre-B cells results in cell cycle impairment, but not in induction of apoptosis.

### Enforced KLF2 Expression Impairs LPS-induced Proliferation and Induces Apoptosis in Splenic B Cells

**Figure 3:**
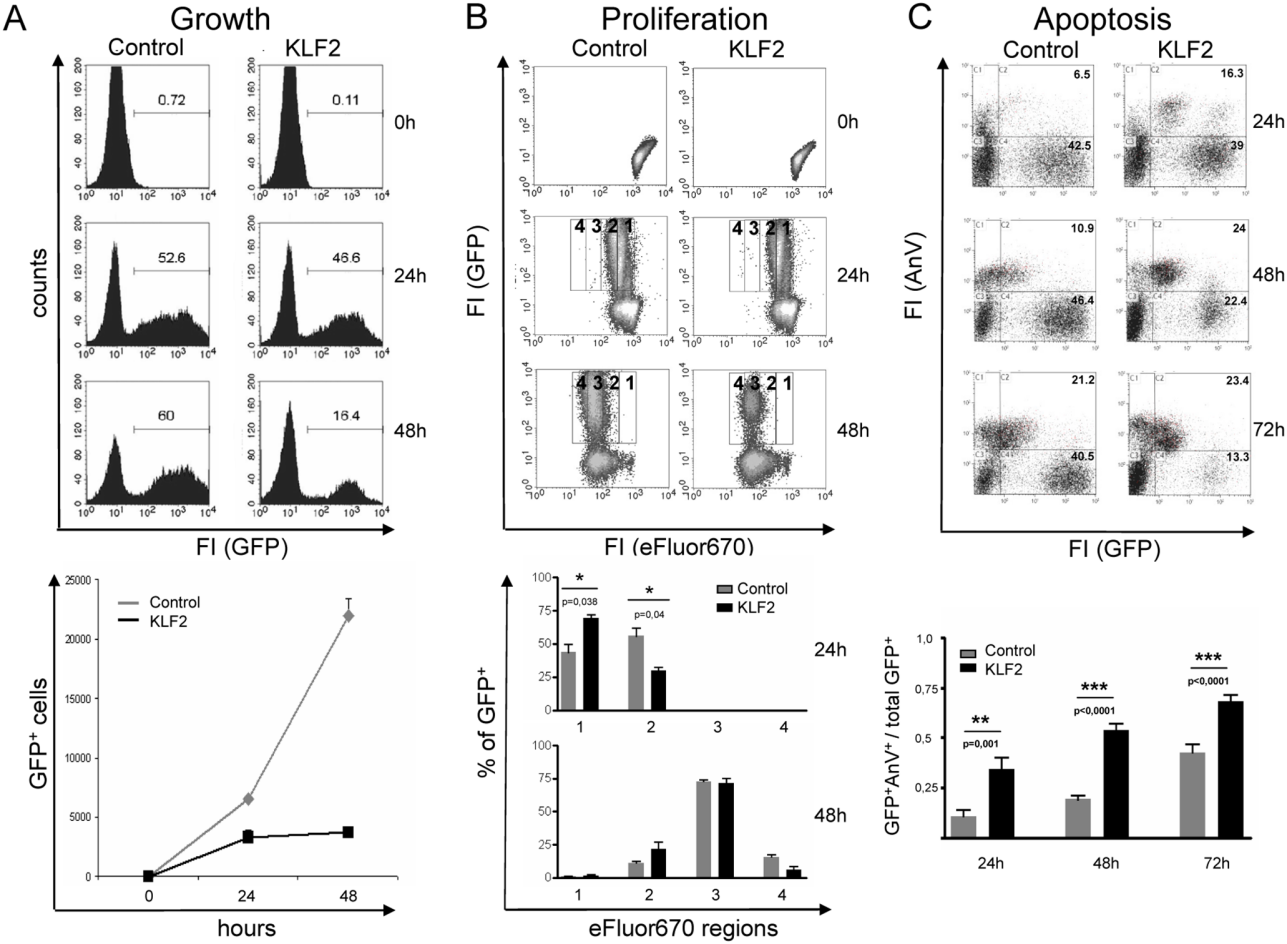
Enforced KLF2 expression impairs proliferation and induces apoptosis in LPS-activated splenic B cells. (**A–C**) Flow cytometric analyses of KLF2- and control-transduced primary CD43^−^ splenic B cells stimulated with LPS. (**A**) Histograms show the frequencies of GFP^+^ (upper panel), whereas the diagram shows the mean numbers ± SD of GFP^+^ cells before (0) and 24 as well as 48 h after infection of one representative of 5 independent experiments measured in triplicate (lower panel). (**B**) eFluor670-labeled GFP^+^ cells where analyzed for the loss of the proliferation dye (regions 1–4) before (0) and 24 as well as 48 h after infection, with only GFP^+^ cells shown (upper panel). Bar diagrams show the mean frequencies ± SEM of GFP^+^ cells in the different regions at the indicated time points and compare control (grey)- and KLF2 (black)-infected cells (n = 3) (lower panel). (**C**) Control- and KLF2-infected cells where analyzed for GFP expression and AnnexinV (AnV) staining. Dot plots of one representative experiment (24 h–72 h) are shown (upper panel). Numbers indicate the frequencies of cells in the respective quadrants. Bar charts (lower panel) show the mean ratios ± SEM of GFP^+^AnnexinV^+^ cells relative to the total number of GFP^+^ cells at the indicated time points (n = 7, i.e., 7 animals/spleens of each group were analyzed).

Others and we have recently shown that KLF2 is present in naive, unstimulated B cells and is rapidly downregulated upon stimulation with LPS and other mitogenic stimuli (Figure S4 in File S1) [17], [19]. To test the hypothesis that KLF2 is indeed responsible for the quiescent phenotype of naive B cells, we restored KLF2 expression in LPS blasts by retroviral infection. Quantification of the KLF2 protein levels in retrovirally transduced B cells compared to endogenous KLF2 in unstimulated B cells showed an approximately 10-fold higher expression (Figure S2B in File S1). If our hypothesis is correct, an impairment of proliferation and an inhibition of activation markers such as AID, IRF4 and Blimp1 should occur. First, we analyzed the cell growth of retrovirally transduced, primary splenic B cells that were stimulated with LPS. As shown in Figure 3A, KLF2-transduced cultures showed a strongly reduced frequency of GFP^+^ cells and constant numbers of GFP^+^ cells (24 and 48 h after infection), whereas control-infected cells showed a constant frequency and an increase in the absolute cell numbers over time, indicating that KLF2-transduced, splenic B cells are impaired in proliferation or become apoptotic. To address this issue, we measured the effect of enforced KLF2 expression on the proliferation of LPS-stimulated B cells by labeling retrovirally transduced splenic B cells with eFluor670 proliferation dye and tracking the loss of fluorescence by flow cytometry over a period of 3 days. As indicated in Figure 3B, there was a significant higher eFluor670 signal after 24 h in KLF2-transduced cells (only 30% in region 2) compared to control cells (56% in region 2). However, after 48 h, no significant changes in eFlour670 fluorescence between control- and KLF2-transduced cells were observed, indicating that enforced expression of KLF2 only slightly impairs LPS-induced proliferation early after stimulation. To analyze whether KLF2 overexpression leads to induction of apoptosis, we stained LPS-stimulated control- or KLF2-infected splenic B cells with AnnexinV. Indeed, we found a highly significant increase in the frequency of GFP^+^/AnnexinV^+^ cells 24 h, 48 h and 72 h after infection with KLF2, as measured by flow cytometry (Figure 3C). In summary, enforced KLF2 expression has only a minor impact on proliferation and leads to the onset of apoptosis in LPS-stimulated splenic B cells.

### KLF2 Represses AID, IRF4 and BLIMP1 Expression in Activated Splenic B Cells

**Figure 4:**
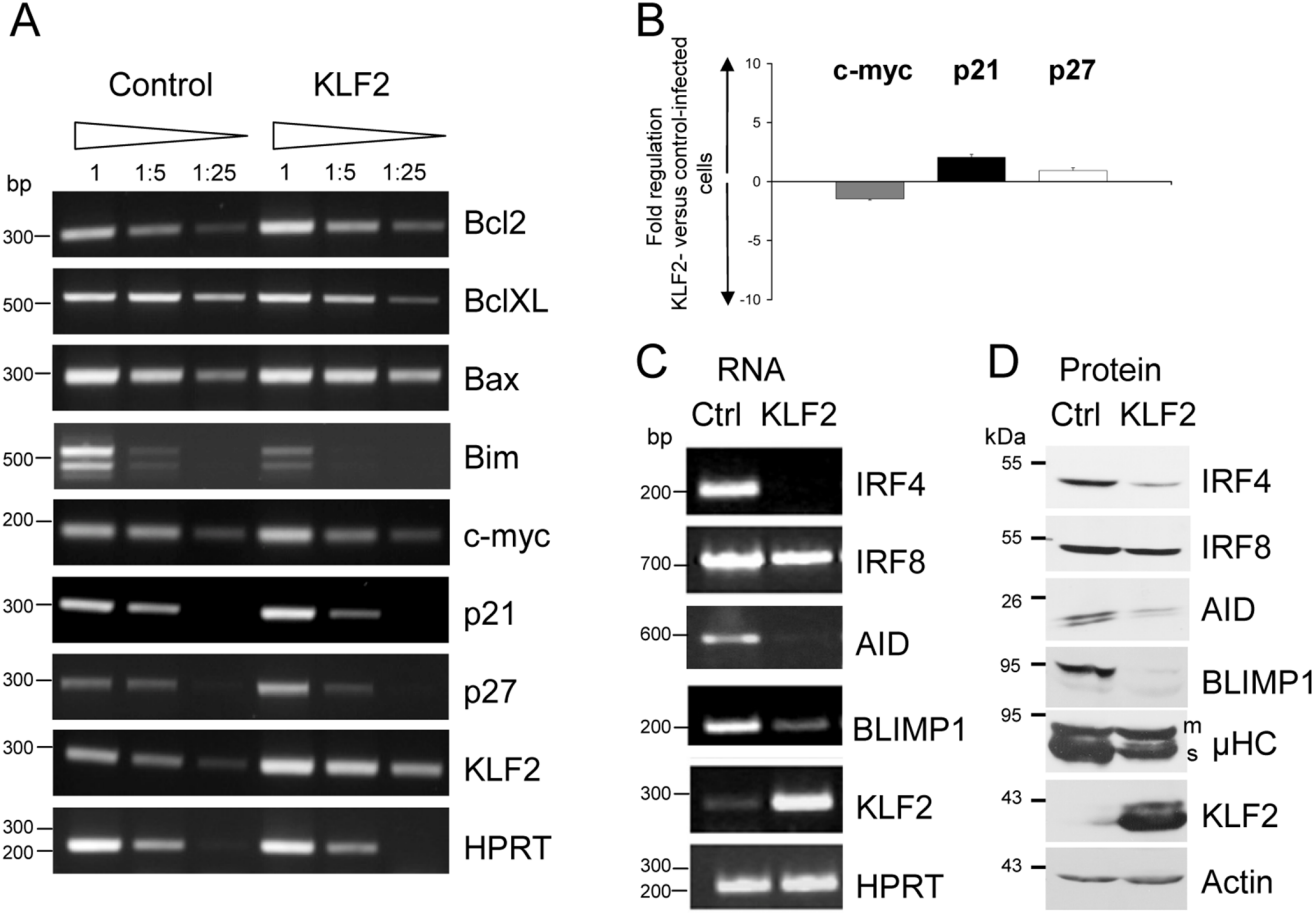
Enforced KLF2 expression blocks the activation of LPS-stimulated primary splenic B cells. (**A, B**) PCR analyses of cell cycle and apoptotic regulators in LPS-stimulated, control- and KLF2-infected, GFP^+^-sorted splenic B cells with transcript specific primers 48 h after infection. HPRT signals control the integrity and amount of cDNA. (**A**) Serial dilutions of the respective cDNAs were used as templates. Fragments of a DNA bp standard are indicated to the left. Representative results of three independent experiments are shown. (**B**) qPCR analyses of c-myc, p21 and p27 transcript levels. Bar graphs indicate the mean fold change (+/− SEM) of the respective transcript in KLF2-infected GFP^+^ compared to control-infected GFP^+^ splenic B cells 48 h after infection. The fold change of transcript abundance was calculated from four independent experiments. RT-PCR (**C**) and Western Blot analyses (**D**) of activation-related molecules in control- (Ctrl) and KLF2-transduced (KLF2), GFP^+^-sorted splenic B cells 48 h after infection. KLF2 abundance served as a control for ectopic expression, whereas Actin abundance served as a control for equal amounts of loaded protein. Fragments of a protein standard, in kDa, are indicated. Representative results of two independent cell sorts with subsequent Western Blot experiments are shown.

We hypothesized that KLF2 is a quiescence factor for naive B cells. If this hypothesis is correct, enforced KLF2 expression should inhibit the upregulation of activation-related molecules such as AID, IRF4 and Blimp1 in LPS cultures. Therefore, we performed RT-PCR analyses of cell cycle regulators, apoptosis regulators and activation-related genes in LPS-stimulated KLF2- and control-transduced splenic B cells that were purified based on GFP expression 48 h after infection (experimental setup: see Figure S5 in File S1). Consistent with our proliferation data, enforced KLF2 expression in splenic B cells did not affect c-myc and p27 transcript levels and only showed an effect on the cell cycle inhibitor p21 (2 fold upregulation), as measured by RT-PCR and qPCR (Figures 4A and 4B). The transcript levels of apoptosis regulators such as Bcl2, BclXL and Bax were not changed, and Bim levels were slightly decreased (Figure 4A). Moreover, we analyzed the effect of transduced KLF2 expression on activation-related genes, such as AID, as well as on regulators of plasmablast differentiation, such as IRF4, IRF8 and BLIMP1 [24], at the transcript and protein levels. As expected, we detected signals for IRF4, IRF8, AID and BLIMP1 transcripts in LPS-stimulated control cells (Figure 4C). However, in KLF2-transduced cells, the abundance of IRF4, AID and BLIMP1 mRNA was strikingly reduced (Figure 4C). There was no change in the abundance of the IRF8, Pax5, Bach2 or Bcl6 transcripts in KLF2-transduced cells (Figure 4C and data not shown). We confirmed KLF2-mediated repression of the protein levels of IRF4, AID and BLIMP1 by Western Blot analysis, as shown in Figure 4D. Moreover, we investigated the protein abundance of the secretory form of µHC (µHC_s_). As shown in Figure 4D, KLF2-transduced cells express less µHC_s_ compared to control cells. In summary, KLF2 interferes with LPS-induced B cell activation by blocking the upregulation of AID, IRF4 and BLIMP1, thereby promoting cell death.

## Discussion

Expression of the pre-BCR in early B cell development is part of a critical checkpoint, where the outcome of VDJ recombination at the IgH locus is monitored [25], [26]. The expression of a functional µHC leads to the assembly of the pre-BCR complex, which is transported to the cell surface. Signals initiated by the pre-BCR complex induce a short, proliferative expansion phase, which results in the amplification of a cell with a functional µHC and, subsequently, in an enlargement of the antibody repertoire. A critical molecular step is the termination of pre-B cell expansion after 2–5 rounds of proliferation [25], [26]. Because deregulation of termination results in hyperproliferation and the generation of pre-B cell leukemia, as demonstrated in BLNK and BLNK/BTK-deficient animals [27], a strict control mechanism must exist. However, the exact molecular mechanisms leading to termination are poorly characterized. We have shown that KLF2 is upregulated as a late target gene of the pre-BCR and is predominantly expressed in small, resting pre-B cells [16]. Because of its specific expression pattern and known anti-proliferative potential [13], [14], we speculate that upregulation of KLF2 during the development of pre-B cells from large, proliferating to small, resting pre-B cells is a key event that heralds the termination of pre-B cell expansion. Indeed, we found that ectopic KLF2 expression resulted in a massive block of proliferation, accompanied by an upregulation of inhibitory proteins, such as p27 and p21, as well as downregulation of c-myc expression. However, in KLF2-deficient animals, an accumulation of pre-B cells could not be observed [17]. KLF4, a closely related transcription factor, is still present in KLF2-deficient pre-B cells. Therefore, we propose that KLF4, due to its overlapping set of target genes, compensates for the loss of KLF2 and terminates pre-B cell proliferation together with Ikaros/Aiolos [28]. To address this issue *in vivo*, analysis of KLF2/KLF4 double knockout mice will provide a deeper insight into the mechanisms by which KLF2 and KLF4 regulate B cell proliferation, migration and quiescence.

Next, we investigated the role of KLF2 in mature B cells. Others and we found the expression of KLF2 in mature, resting splenic B cells. Upon stimulation mimicking T-independent activation (LPS) or T-dependent activation (CD40/IL4/IgM), KLF2 was downregulated at the transcript as well as protein level (Figure S4 in File S1) [17], [29], suggesting that KLF2 is a potent quiescence factor and its downregulation is a prerequisite for B cell activation as well as B cell proliferation. Here, we indeed identified KLF2 as a negative regulator of B cell activation and plasmablast differentiation. We showed that KLF2 exerts its role as a quiescence factor by repressing IRF4, AID and BLIMP1 expression. This is underlined by the observation that KLF2-transduced cells produce less secretory µHC_s_.

In summary, we observed two modes of action for KLF2. Pre-B cells react to premature KLF2 expression with impaired proliferation, which reflects the termination of pre-B cell expansion that is observed *in vivo* [24]. In splenic B cells, enforced KLF2 expression results in an impairment of activation, rather than a blocking of the cell cycle (as shown by downregulation of AID, IRF4 and Blimp1). By transducing LPS-stimulated B cells with KLF2, we attempted to reprogram the cells from an activated to a non-activated, resting state. However, LPS-induced proliferation was not affected. Therefore, we speculate that LPS-induced signals cannot be completely reversed by KLF2 because cells have passed a point of no return. We propose that conflicting signals (i.e., activating signals initiated by LPS and inhibitory signals delivered by KLF2) lead to cell death. Hence, we conclude that KLF2 is critical for terminating pre-B cell clonal expansion and for maintaining a quiescent B cell phenotype by repressing genes involved in B cell activation and plasmablast differentiation.

## Materials and Methods

### Animals and Ethics Statement

dTg mice (Rag2^−/−^ tTA^+^ TetO-Sp6-µHC mice) (Figure S1A in File S1) [22] and C57Bl/6 mice (Janvier, France) were maintained under pathogen-free conditions in the IVC animal facility of the Franz-Penzoldt Centre (Erlangen, Germany). Transgenic animals were genotyped by PCR using tail biopsies. All animal experiments were performed in accordance with the German Law on Care and Use of Laboratory Animals. Euthanasia and organ preparation were approved by the local authorities (Landratsamt Erlangen-Hoechstadt, Veterinaeramt Erlangen, Germany (File number TS 4/07).

### Antibodies

Anti-KLF2-rabbit serum was generated by immunizing rabbits with a KLF2-specific peptide [16]. Rabbit anti-Actin antibodies were purchased from Sigma (Sigma, Deisenhofen, Germany). Goat anti-mouse IRF8 and IRF4 Abs were from Santa Cruz, and rat anti-BLIMP1 was from Cell Signaling Technology (CST, Danvers, MA). To detect AID, biotinylated anti-AID clone 94.16 [30] followed by Streptavidin-HRP (Amdex, Thermo Scientific, Waltham, MA) was used. HRP goat anti-rabbit antibodies and HRP goat anti-mouse IgM were purchased from Southern Biotechnologies (Birmingham, AL). HRP donkey anti-goat IgG was from Santa Cruz (Santa Cruz, CA), and HRP goat anti-rat IgG was obtained from Jackson ImmunoResearch (Newmarket, UK).

### Cell Sorting and Cell Culture

Pro-B cells were isolated by magnetic cell sorting using CD19 magnetic beads (Miltenyi Biotech, Bergisch-Gladbach, Germany) from the bone marrow of Rag2^−/−^ dTg mice that received 200 µg/ml Tetracycline (Tet) with the drinking water for 7 days. CD19-positive cells were cultured with IL-7, as previously described [16], [23], in the presence or absence of Tet (100 ng/ml). CD43-negative splenic B cells were isolated from C57Bl/6 mice by CD43 depletion using MACS technology (Miltenyi, Bergisch-Gladbach, Germany) to a purity of approximately 95%. Purified splenic B cells were stimulated with 10 µg/ml LPS (Sigma, Deisenhofen, Germany), anti-IgM (clone b7.6; 10 µg/ml) or a combination of anti-CD40 (clone FGK; 10 µg/ml) and 100 U/ml recombinant IL4 (Miltenyi Biotech, Bergisch-Gladbach, Germany). GFP-positive cells were sorted to a purity >98% using a MoFlo cell sorter (Dako Cytomation, Glostrup, Denmark).

### Retroviral Infection of Primary B Lymphoid Cells

To generate retroviral particles, Phoenix-E cells were transfected with pBMN-KLF2-IRES-GFP or pBMN-IRES-GFP constructs (Figure S2A in File S1), and virus-containing supernatants were collected 48 h and 72 h after transfection. Prior to infection, primary dTg CD19^+^ lymphoid cells were cultured for 24 h in RPMI1640, 10% FCS (Invitrogen, Karlsruhe, Germany) with IL-7 in the absence of Tet. Primary, CD43-negative splenic B cells were cultured for 24 h in RPMI1640, 10% FCS in the presence of 10 µg/ml LPS (Sigma, Deisenhofen, Germany). dTg pre-B cells as well as splenic B cells were infected with viral supernatants mixed with 10% fresh medium and 0.1% polybrene. Cells were centrifuged for 3.5 h at 3000 rpm at 33°C, virus-containing supernatants were removed, and fresh medium was added to the cells.

### Proliferation, Flow Count and Apoptosis Assays

Immediately after infection, pre-B as well as splenic B cells were plated, in triplicate, in wells of a 96-well plate at a defined cell density. After infection, the cells were harvested at the indicated time points and mixed with a defined volume of fluorosphere flow count beads (Beckman-Coulter, Krefeld, Germany). The number of living cells was determined by FSC/SSC characteristics and analyzed by flow cytometry using a FACSCalibur and the CellQuest software (BD Biosciences, San Diego, CA). For proliferation analyses, cells were labeled with eFluor670 Cell Proliferation Dye (eBioscience, San Diego, CA) according to the manufacturer’s protocol. Immediately after labeling, eFluor670 fluorescence was analyzed by flow cytometry, adjusted at day 0 and measured with the same settings from day 1 to day 3. AnnexinV-APC (eBioscience, San Diego, CA) staining was performed according to the manufacturer’s protocol. All FACS analyses were performed using a FACSCalibur (BD Biosciences, San Diego, CA) or Gallios flow cytometer (Beckman-Coulter, Krefeld Germany). All data analyses were performed using the CellQuest (BD Biosciences, San Diego, CA) or Kaluza (Beckman-Coulter, Krefeld, Germany) software.

### RNA Isolation, RT-PCR and qPCR

Total RNA was isolated according to the RNeasy Kit protocol (Qiagen, Hilden, Germany) and was reversely transcribed into cDNA using the Superscript-RT II Kit (Invitrogen, Karlsruhe, Germany). cDNA was amplified by PCR using transcript-specific primers (Table S1 in File S1). SYBR Green qPCR was performed on an Applied Biosystems GeneAmp 7300 system (Applied Biosystems, Darmstadt, Germany) using the ABsolute SYBR Green ROX Mix (Thermo Scientific, Waltham, MA) and transcript-specific primers (Table S1 in File S1). qPCR reactions were performed in triplicate. Ct values of the c-myc, p21 and p27 transcripts were normalized to beta-Actin. To calculate the fold change, ΔΔCt values of four independent cDNAs obtained from independent infection/GFP sorting experiments were used.

### Western Blot Analysis

Cells were lysed in NET/TritonX-100 lysis buffer, and extracts were resolved on polyacrylamide-protein gels as previously described [31]. Proteins were then electroblotted onto nitrocellulose membranes and stained with the respective antibodies.

### Statistical Analysis

All statistical analyses were performed using the GraphPad Prism-Software (GraphPad software, La Jolla, CA). Statistical significance was calculated by a paired t test using two-tailed analysis with a 95% confidence interval. Significance is shown as p<0.05*, p<0.01** and p<0.001***.

## Supporting Information

File S1 Combined Supporting Information File containing Figures S1–S5 and Table S1.
(PDF)
