# Supplementary material for: KLF2– A Negative Regulator of Pre-B Cell Clonal Expansion and B Cell Activation
Source: PLoS One. 2014 May 29;9(5):e97953. doi: 10.1371/journal.pone.0097953 (PMC4038547; doi:10.1371/journal.pone.0097953)
Supplement: File S1 — Combined Supporting Information File containing Figures S1–S5 and Table S1. (PDF) [file pone.0097953.s001.pdf]

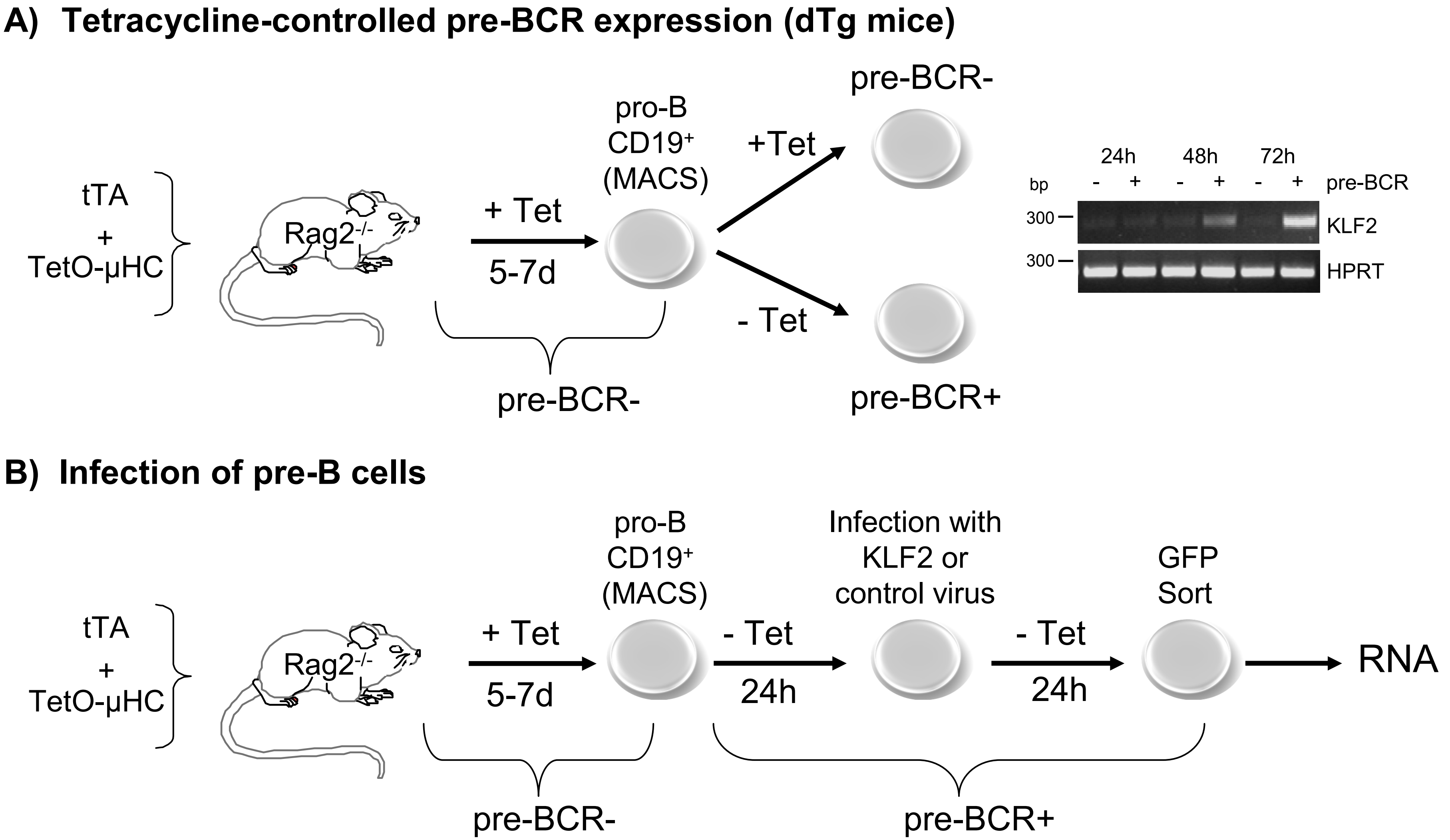

Figure S1: Tetracycline-controlled pre-BCR expression.

A) Schematic overview of tetracycline-dependent expression of the pre-BCR. dTg (Rag2$^{-/-}$ / tTA / TetO-μHC) animals receive tetracycline (Tet) with the drinking water. Transgenic μHC and subsequent pre-BCR expression is turned off and B cell development is arrested at the pro-B cell stage. Upon sorting of CD19-positive pro-B cells, cells are cultured either in the absence or presence of Tet. In the absence of Tet, pre-BCR expression is turned on. PCR analysis of KLF2 transcripts in response to pre-BCR induction (modified after Schuh et al., 2008). **B)** Schematic timeline of retroviral transduction of dTg pre-B cells with subsequent GFP sort. Cells were cultured in the absence of Tet to turn on pre-BCR expression.

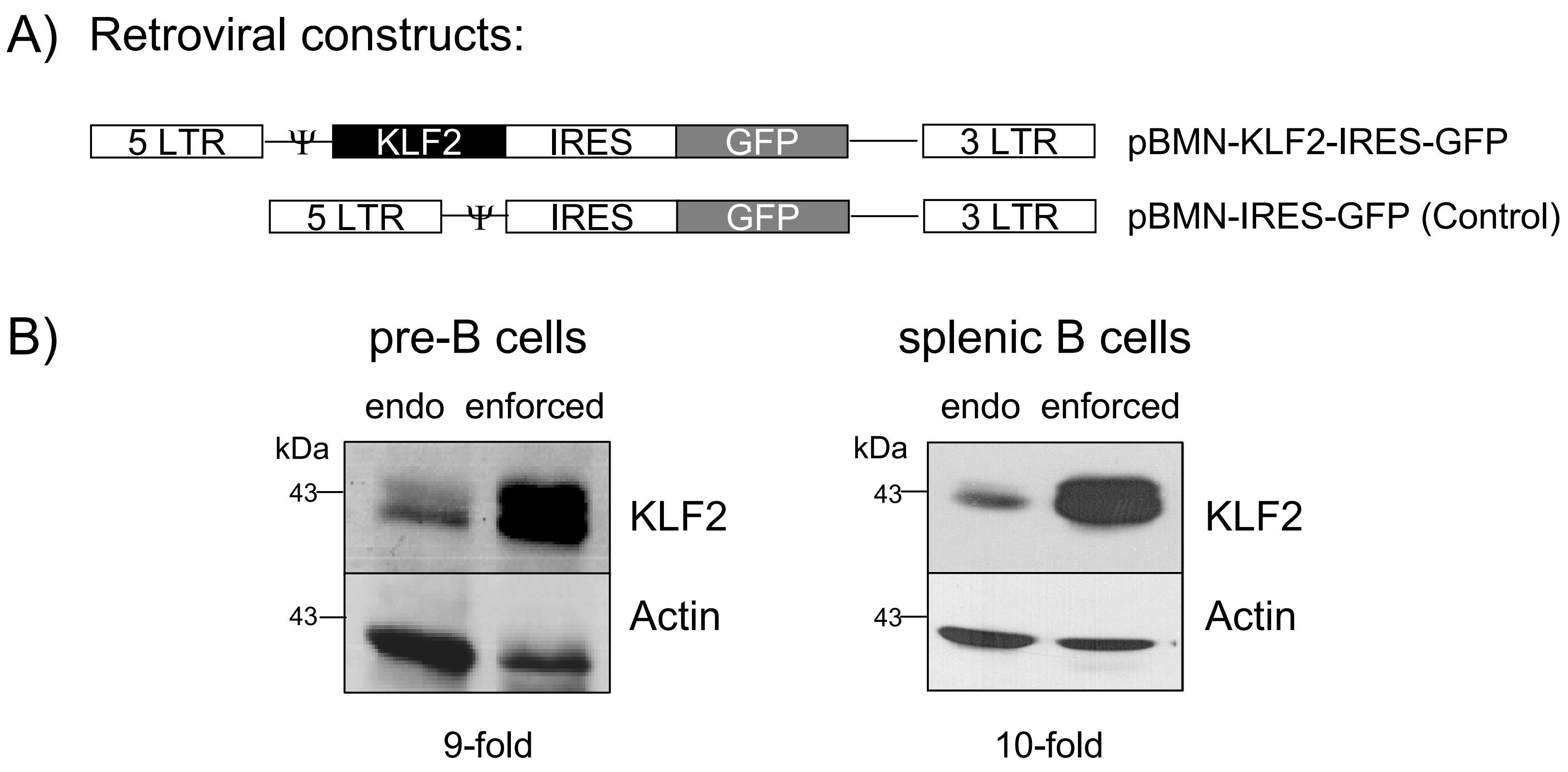

Figure S2: Enforced KLF2 expression by retroviral infection of primary B cells.

A) Schematic structure of the retroviral constructs. pBMN vectors contain LTRs derived from Moloney Mouse Leukemia Virus and an IRES (internal ribosome entry site) sequence. For generation of pBMN-KLF2-IRES-GFP the cDNA sequence of murine KLF2 was cloned into the pBMN-IRES-GFP vector. **B)** Western Blot analyses of endogenous (endo) and enforced KLF2 protein levels in dTg pre-B cells and splenic $CD43^-$ B cells. For detection of enforced KLF2 expression, GFP^+ pre-B cells were sorted 24 h post infection and GFP^+ splenic B cells were sorted 48 h after retroviral transduction. For detection of endogenous KLF2 in dTg pre-B cells, cells were lysed after 72 h in culture in the absence of Tet. For endogenous KLF2 in splenic B cells, freshly isolated $CD43^-$ splenic B cells were lysed. KLF2 signal intensities as well as Actin signal intensities were measured with ImageJ software. The signal intensity ratio of enforced to endogenous KLF2 was normalized to Actin signals and resulted in an approximate 9-fold and 10-fold enforced expression of KLF2 in pre-B cells and splenic B cells, respectively.

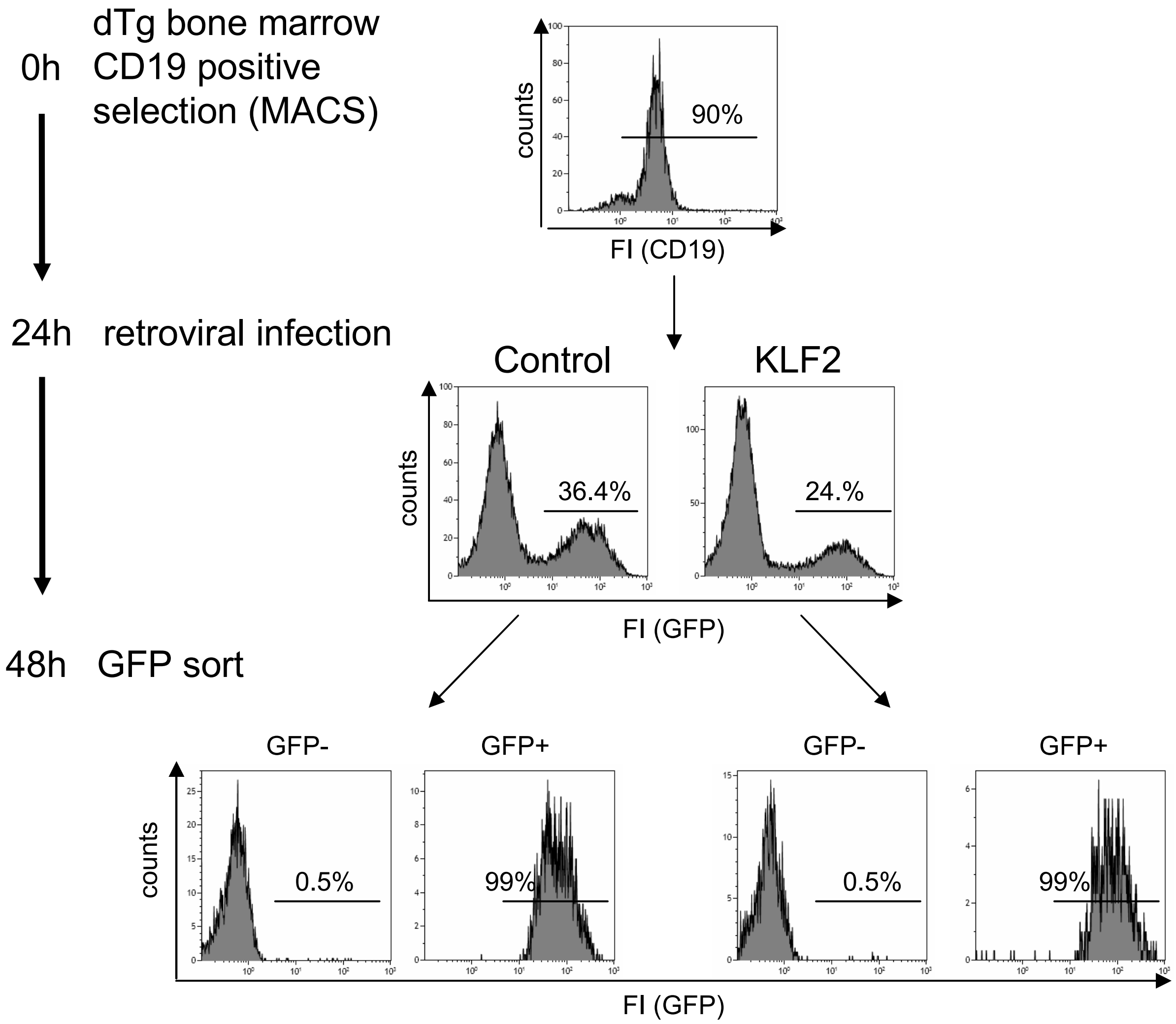

Figure S3: Schematic overview of isolation, infection and GFP sorting of primary CD19+ dTg precursor B cells. CD19+ pro-B cells were purified from the bone marrow of dTg animals by CD19 positive selection using MACS technology. Purity of isolated cells was assessed by flow cytometry using CD19 staining (upper panel). After purification CD19+ cells were cultured in the presence of IL7 and without Tet (pre-BCR expression on) and infected after 24 h with either KLF2 or control viral supernatants. Infection efficiency was measured by flow cytometry (middle panel). 24 h after infection (48 h after isolation) GFP positive and negative fractions were sorted using a MoFlo cell sorter. Purities of GFP negative and positive fractions were determined by flow cytometry and were routinely >99% (lower panel).

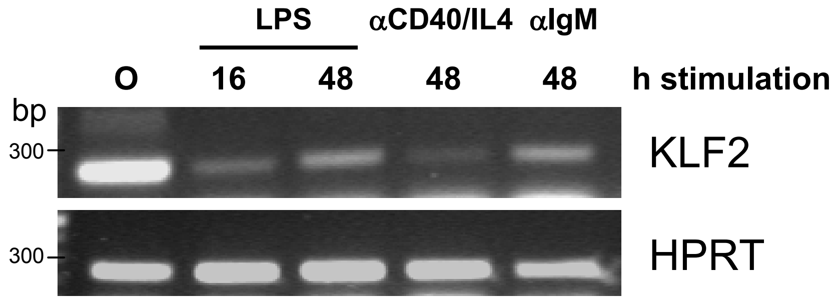

Figure S4: Regulation of KLF2 transcripts upon activation of CD43⁻ splenic B cells.
RT-PCR analyses of KLF2 transcripts in naive, resting B cells (0 h) and activated B cells (with either LPS, anti-CD40/IL4 or anti-IgM). HPRT served as a control for RNA/cDNA integrity and for equal loading.

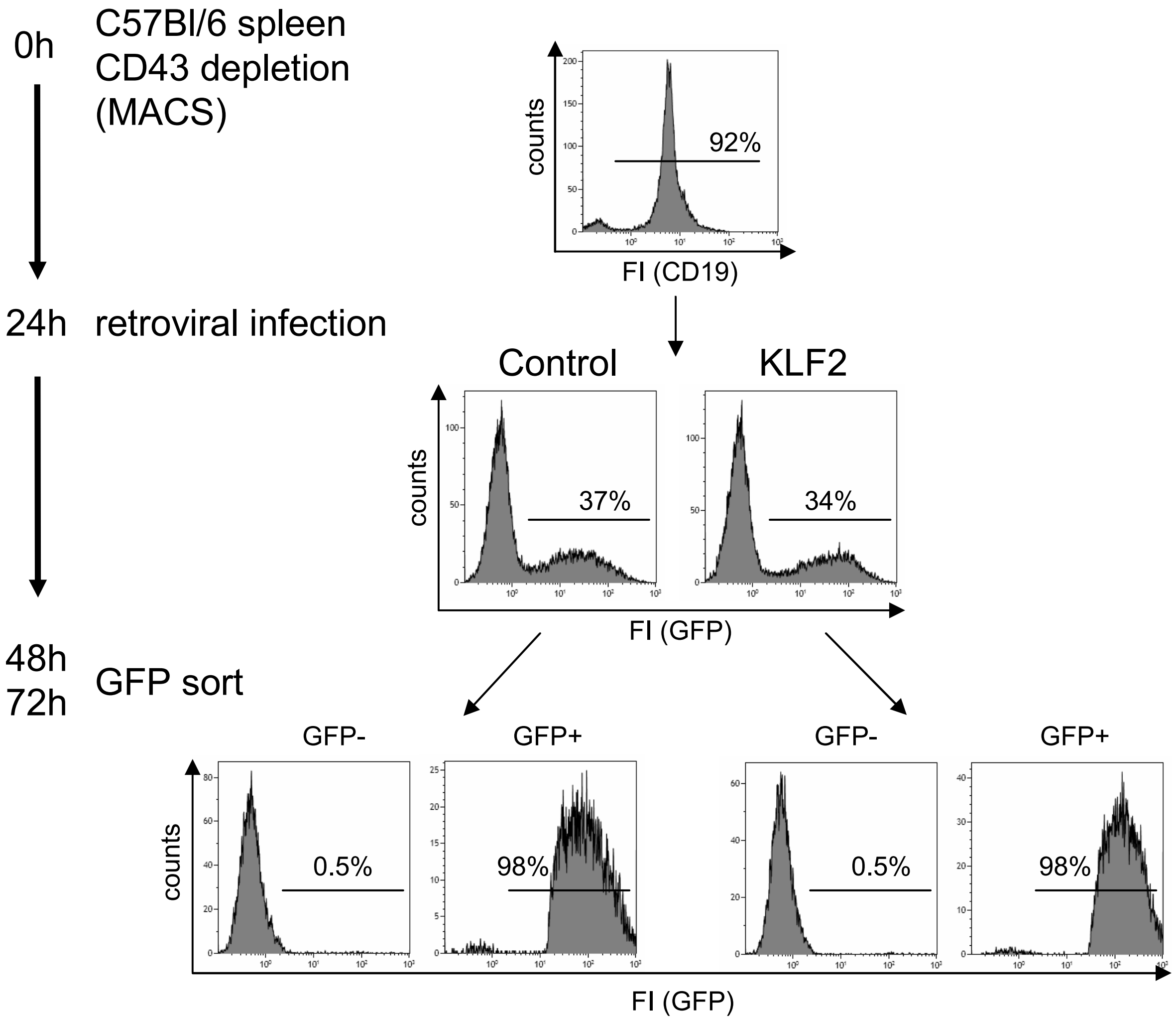

Figure S5: Schematic overview of isolation, infection and GFP sorting of primary splenic CD43$^-$ B cells. Splenic B cells were purified by CD43 depletion using MACS technology. Purity of isolated cells was assessed by flow cytometry using CD19 staining (upper panel). After purification B cells were cultured in the presence of LPS and infected 24 h later with either KLF2 or control viral supernatants. Infection efficiency was measured by flow cytometry (middle panel). 24 h and/or 48 h after infection (48 h and 72 h after isolation, respectively) GFP positive and negative fractions were sorted using a MoFlo cell sorter. Purities of GFP negative and positive fractions were determined by flow cytometry and were routinely >98% (lower panel).

Primer name	Sequence (5' – 3') forward	Sequence (5' – 3') reverse	Amplicon (bp)
AID	ATGGACAGCCTTCTGATGAAG	TCAAAATCCCAACATACGAAATG	597
Bax	AGACACCTGAGCTGACCTTG	CTTGGATCCAGACAAGCAGC	339
Bcl2	TCGCTACCGTCGTGACTTC	AAACAGAGGTCGCATGCTG	313
BclXL	TGGTCGACTTTCTCTCCTAC	GAGATCCACAAAAGTGTCCC	557
Beta-Actin (qPCR)	CGGTTCCGATGCCCTGAGGCTCTT	CGTCACACTTCATGATGGAATTGA	100
Bim	ATGGCCAAGCAACCTTCTGA	TCAATGCCTTCTCCATACCA	591, 423, 333 (3 transcripts)
Blimp1	ATGAGAGAGGCTTATCTCAGATG	GGGTGGTCGTTCACTATGTATG	211
c-myc (qPCR)	TCTCCACTCACCAGCACAACTACG	ATCTGCTTCAGGACCCT	103
c-myc	GGGCCAGCCCTGAGCCCCTAGTGC	ATGGAGATGAGCCCGACTCCGACC	156
HPRT	GCTGGTGAAAAGGACCTCT	CACAGGACTAGAACACCTGC	249
IRF4	ATGAACTTGGAGACGGGCAG	GAGGATCTGGCTTGTCGATC	268
IRF8	CAGGATGTGTGACCGGAACG	GCCCATACAACTTAGGCAGC	728
KLF2	ATGGCGCTCAGCGAGCCTAT	AGCAGCTCTGTTCCCAGGCT	284
p21 (qPCR)	GCAGATCCACAGCGATATCC	CAACTGCTCACTGTCCACGG	130
p21	AGTGTGCCGTTGTCTCTTCG	ACACCAGAGTGCAAGACAGC	311
p27 (qPCR)	AAGGGCCAACAGAACAGAAG	GGATGTCCATTCAATGGAGTC	218
p27	TCGCAGAACTTCGAAGAGG	TGACTCGCTTCTTCCATATCC	300

Table S1: Primers used for RT-PCR and qPCR analyses
